# Theranostics in the vasculature: bioeffects of ultrasound and microbubbles to induce vascular shutdown

**DOI:** 10.7150/thno.70372

**Published:** 2023-07-14

**Authors:** Frederic Padilla, Jacqueline Brenner, Francesco Prada, Alexander L Klibanov

**Affiliations:** 1 Focused Ultrasound Foundation, Charlottesville, VA 22903, USA.; 2 Department of Radiology, University of Virginia School of Medicine, Charlottesville, VA 22908, USA.; 3 Ultrasound Neuroimaging and Therapy Lab, Fondazione IRCCS Istituto Neurologico C. Besta, Milan, Italy.; 4 Department of Neurological Surgery, University of Virginia School of Medicine, Charlottesville, VA, USA.; 5 Department of Biomedical Engineering, University of Virginia, Charlottesville, VA 22908, USA.; 6 Cardiovascular Division, Department of Medicine, University of Virginia, Charlottesville, VA 22908, USA.

**Keywords:** antivascular therapy, ultrasound, cavitation, microbubbles, drug delivery, tumor therapy

## Abstract

Ultrasound-triggered microbubbles destruction leading to vascular shutdown have resulted in preclinical studies in tumor growth delay or inhibition, lesion formation, radio-sensitization and modulation of the immune micro-environment. Antivascular ultrasound aims to be developed as a focal, targeted, non-invasive, mechanical and non-thermal treatment, alone or in combination with other treatments, and this review positions these treatments among the wider therapeutic ultrasound domain. Antivascular effects have been reported for a wide range of ultrasound exposure conditions, and evidence points to a prominent role of cavitation as the main mechanism. At relatively low peak negative acoustic pressure, predominantly non-inertial cavitation is most likely induced, while higher peak negative pressures lead to inertial cavitation and bubbles collapse. Resulting bioeffects start with inflammation and/or loose opening of the endothelial lining of the vessel. The latter causes vascular access of tissue factor, leading to platelet aggregation, and consequent clotting. Alternatively, endothelium damage exposes subendothelial collagen layer, leading to rapid adhesion and aggregation of platelets and clotting. In a pilot clinical trial, a prevalence of tumor response was observed in patients receiving ultrasound-triggered microbubble destruction along with transarterial radioembolization. Two ongoing clinical trials are assessing the effectiveness of ultrasound-stimulated microbubble treatment to enhance radiation effects in cancer patients. Clinical translation of antivascular ultrasound/microbubble approach may thus be forthcoming.

## 1. Introduction

Ultrasound microbubbles (MB) have been investigated for many years for their potential to promote the uptake of drugs into the tumor tissue by exploiting their ability to enhance vascular permeability 1. Clinical studies are currently exploiting this mechanism to transiently open the blood brain barrier (BBB) for drug delivery 2,3. Recent publications using either implantable probe 4 or an MRI guided external multi-elements array 5,6 support the generalizability of therapeutic use of ultrasound-activated microbubbles for neuro-oncological applications. These ongoing clinical studies demonstrate the safety and feasibility of BBB opening, with potential for improved delivery of drugs.

These drug delivery schemes rely on bioeffects following stable, non-inertial cavitation of the microbubbles to induce transient openings of endothelial cell junctions 7. The more violent effects that can be induced through inertial cavitation, or the violent collapse of the microbubbles 8, have initially been considered as undesirable. It has been reported for more than 30 years that ultrasound can induce capillaries rupture *in vivo* when treatments involve cavitation such as lithotripsy 9-12, or when driving microbubbles at sufficiently high intensity 13-21, and that even large vessel can be damaged by ultrasound driven microbubbles 22. It has also been reported that those damages can occur even under diagnostic ultrasound exposures 20,23,24, with in general the pressure amplitude and the MB dose governing the extent of produced bioeffects 25. Only relatively recently the bioeffects induced by ultrasound exposure of microbubbles have been purposely looked at specifically as therapeutic effects. They have been studied to inhibit tumor growth through a shutdown of tumor blood flow, the induction of non-thermal lesion, or the radiosensitization of tumors when combined with radiotherapy. These anti-vascular effects of ultrasound microbubbles have been reviewed in 26-28.

To our knowledge, three clinical trials are currently being conducted to investigate therapeutic bioeffects induced by microbubbles destruction. The preliminary efficacy results of a pilot clinical trial, using ultrasound-triggered microbubble destruction for radio-sensitization during radioembolization for the treatment of liver cancers, showed a greater prevalence of tumor response in patients receiving both microbubbles and transarterial radioembolization 29. Two other ongoing clinical trials will assess the effectiveness of ultrasound stimulated microbubble treatment to enhance radiation effects in patients with chest-wall and locally-advanced breast cancer (NCT04431674), or head and neck cancers (NCT04431648).

Various mechanisms have been proposed as the source of therapeutic bioeffects associated with microbubbles destruction by ultrasound. Large range of treatment parameters, including microbubbles doses and sonication regimens, have been reported. The relevance of preclinical findings for the desirable parameters of the respective clinical studies, for the most part, remains an open question.

This review discusses the proposed mechanisms that may explain the observed bioeffects, and tries to elucidate the doses, in terms of microbubbles amount or concentration, and therapeutic ultrasound parameters such as treatment duration or peak acoustic pressure, that are required to induce vascular bioeffects and to promote clinical applications.

The issue of the dose is discussed with regards to possible need for the comparison of the concentrations and quantities of the microbubbles across the studies already published, and relevant metrics to quantify other treatment parameters and responses, such as cavitation monitoring, and ultrasound pulse repetition frequency.

## 2. Interactions of microbubbles with ultrasound

Before diving into more detailed descriptions of how bioeffects are induced by combined treatment with ultrasound and microbubbles, it is worth reviewing the properties of microbubbles, the ultrasound treatment parameters space and discussing the general mechanism behind the interaction.

### Ultrasound microbubbles: Properties, characterization, and recommended dosage

**Figure 1:**
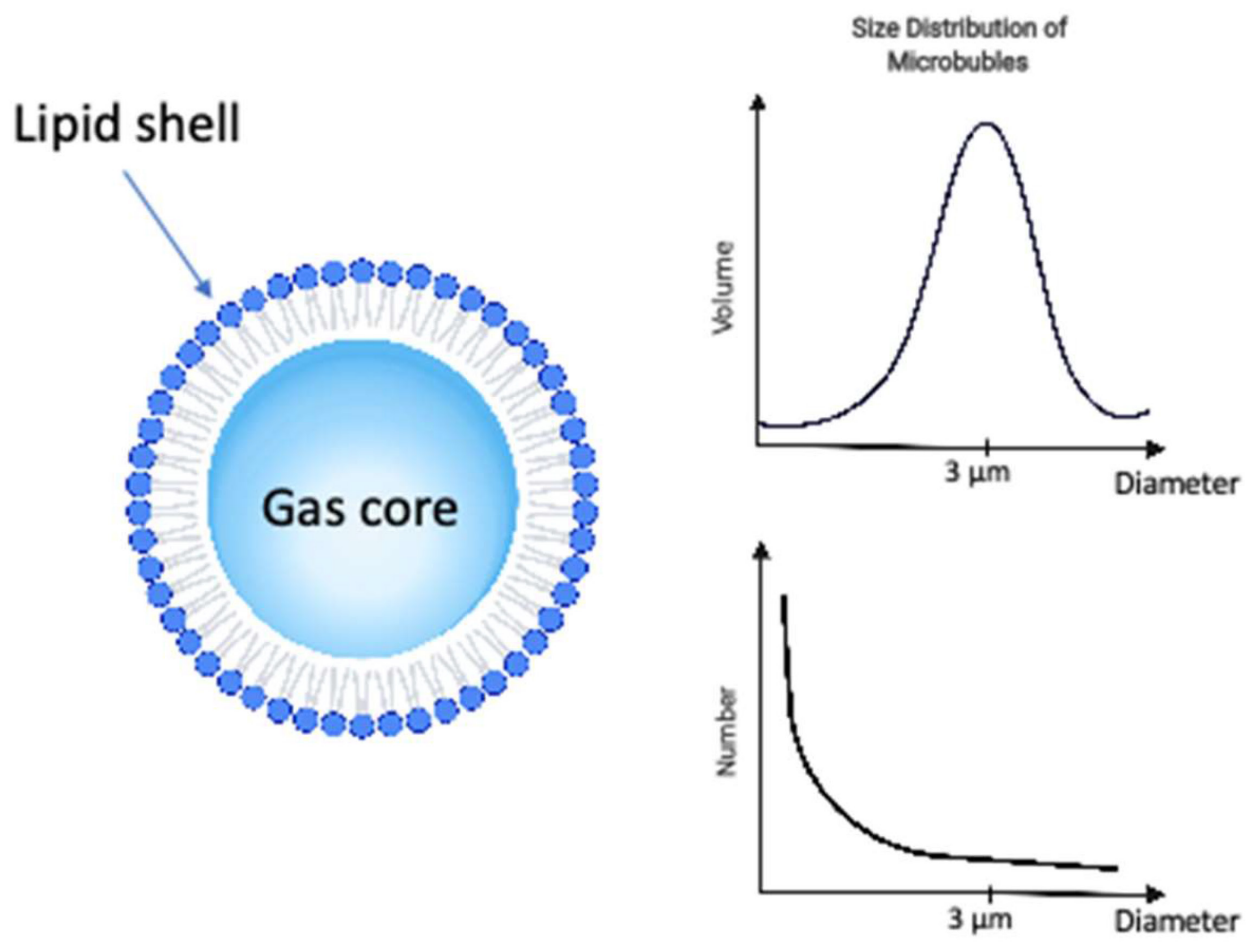
The ultrasound microbubble is composed by a gas core, stabilized by a shell of phospholipid or albumin for the formulations clinically approved for ultrasound contrast imaging. It can be characterized by the composition of its shell, gas core and size distribution among other properties. The distribution microbubbles sizes can be characterized either by the size distribution in volume (top right) or size distribution in number (bottom right), both providing complimentary information.

The microbubbles, or ultrasound contrast agents (Figure 1), used in combination with ultrasound to induce vascular bioeffects, are micro-sized gas bubbles coated with a stabilizing layer to provide a compromise between longevity and echogenicity 30,31.

There are currently three FDA approved contrast agents, Lumason, also marketed as SonoVue in Europe (Bracco Imaging SpA, Colleretto Giacosa, Italy) composed of a phospholipid monolayer shell and sulfur hexafluoride gas core; Definity (Lantheus Medical Imaging, North Billerica, MA) composed of a phospholipid shell and perflutren gas (C_3_F_8_, Octafluoropropane); and Optison (GE Healthcare AS, Oslo, Norway) composed with an albumin shell and perflutren gas (C_3_F_8_, Octafluoropropane). In Japan and Korea, a fourth contrast agent is available, Sonazoid (GE Healthcare Inc., Princeton, NJ, USA), which has perfluorobutane gas core and phosphatidylserine shell coating.

The physicochemical characterization of ultrasound agents has been reviewed in the literature (see e.g., 32), and is summarized in Table 1. The mean diameter in volume is similar for all 3 agents around 8 µm, whereas the diameter in number is slightly higher for Optison (3µm) than for Lumason (1.9µm) or Definity (1.22µm). There is a noticeable disparity between the agents in terms of initial concentration, with Definity more concentrated (84x10^8^ MB/mL) compared to Optison (7.3x10^8^ MB/mL) or Lumason (3.4x10^8^ MB/mL). Caution must be taken when comparing these numbers for different agents, however, as there have been discrepancies in the literature or in the official prescribing information sheets. For instance, in a manuscript that assessed the size distribution of Definity microbubbles^32^, it was reported that Definity particle concentration was 1.3x10^10^ MB/mL. The measurement in the latter study was performed with Coulter counter, in the 0.48-12 um range, so that submicrometer particles could be detected, and dominated the number distribution by an order of magnitude. The prescribing information sheet, “Package Insert”, for Definity microbubbles lists particle number concentration as 1.2x10^10^, very close to what was reported in 33. It is highly likely that particle counting to generate the latter number takes submicron particles into account. Unfortunately, the detailed information on the specific methods and apparatus used to count the microbubbles are not always included within the FDA prescribing information. The presence of very small microbubbles may not be of interest for imaging in the MHz frequency range used in clinics but may be a beneficial source of cavitation nuclei to induce cavitation-related bioeffects.

In terms of a typical injected dose for imaging, there are also significant differences between the agents, both for the injected gas volume (1.65µg/kg for Optison and Definity, 0.77 µg/kg for Lumason) and for the total number oof microbubbles/animal weight or MB/kg (12.6x10^6^ MB/kg for Definity, 5.6x10^6^ MB/kg for Lumason and Optison). Doses of microbubbles for clinical imaging have been reviewed by Hyvelin et al. 32. The values for the full clinical doses for bolus injection of the aqueous volume of contrast medium are 34 µL/kg (2.4 mL/bolus for a 70-kg person) for SonoVue 34, 3 µL/kg (0.2 mL/bolus for a 70-kg person) for Definity 35, and 15 µL/kg, (1 mL/bolus for a 70-kg person) for Optison 36. Doses for clinical imaging with microbubbles infusion may differ, and recommended doses are specified in FDA prescribing information notice only for Definity: 1.3mL, with a rate of infusion initiated at 4.0 mL/minute and not to exceed 10 mL/minute. We should note that the use of the microbubble carrier aqueous medium for dosage should always be accompanied with the particle number concentration, as well as particle gas volume.

### Ultrasound treatment parameters

Ultrasound waves generated by a transducer are mechanical pressures waves. The properties of these pressure waves and of the ultrasound field will govern the dynamics of the microbubbles' behaviors. An ultrasound treatment sequence is defined by its center frequency, peak rarefractional pressure, also known as peak negative acoustic pressure (PNP), the pulse length (or number of cycles), the repetition frequency of the pulse (or pulse repetition frequency PRF) and the total insonification time. Treatment area can be highly localized, with treatment dimensions down to a few millimeters in diameter when using focused transducer; or can be larger when using focused transducer of a few centimeters in dimensions. The microbubble dynamics will vary depending on several factors including these ultrasound pressure field and exposure conditions, but also depending on the microbubble population characteristics, the local microenvironment and tissue type.

### Proposed physical mechanism of ultrasound-microbubbles interaction: Cavitation

**Figure 2:**
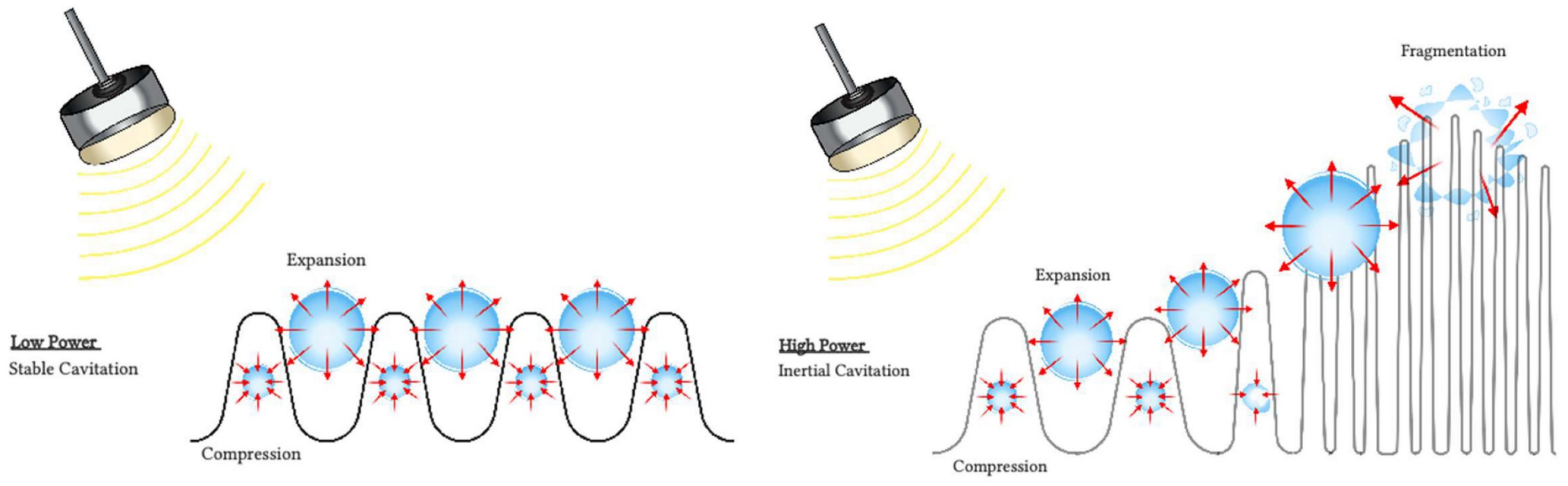
Interactions of microbubbles with ultrasound. During non-inertial cavitation (left), the microbubbles radius oscillates under alternative positive and negative pressures but remains mainly stable overtime. During inertial cavitation (right), the microbubbles radius increases overtime until the microbubble collapses.

Several mechanisms have been proposed to explain observed blood flow disruption in response to insonification of intravascular microbubbles with ultrasound. Although the precise mechanisms responsible for antivascular effects remain uncertain, one prominent candidate is cavitation (Figure 2).

Cavitation refers to the oscillations of microbubbles under the compressional and rarefactional phases of an ultrasound pressure wave 37. Cavitation can be described as stable or transient. Stable cavitation is usually associated with non-inertial cavitation, where the ultrasound field governs the dynamics of the microbubbles. The microbubbles oscillate until they collapse or fragment 38, or their gas dissolves into the surrounding fluid 39. Transient cavitation is generally associated with inertial cavitation, where microbubbles will undergo an unstable expansion phase leading to a rapid collapse dominated by the inertia of the surrounding fluid. Each type of cavitation can induce distinct vascular bioeffects, and will be influenced by the ultrasound field, the microbubbles composition, distribution and concentration and the vascular environment the microbubbles circulate in 40,41.

Inertial cavitation is thought to be a main mechanism of action of microbubbles for vascular damage through violent bubble collapse. It can produce localized regions of high shear stress and temperatures, jetting 42 and an overexpansion or invagination of microvessels 43. Microvascular damages including hemorrhage, edema and endothelial cell damage and apoptosis 22,40,44,45 have been reported in several tissue types such as tumors and brain. Increased cavitation dose has been linked to increased area of extravasation of red blood cells in the cremaster muscle of rats 46. Several reports of the presence of inertial cavitation when treating tumors (47,48 and Table 3) or lesioning normal brain tissue (49 and Table 4) are consistent with the hypothesis that these phenomena are dominantly associated with inertial cavitation.

Using passive cavitation detector to monitor the onset of inertial cavitation, several studies reported that in the absence of inertial cavitation, vascular shutdowns were not observed (Table 3). Whether these inertial cavitation-induced bioeffects are purely mechanical or thermal in nature cannot be determined from these studies, since it cannot be ruled out that localized temperature elevations occurring in the immediate vicinity of collapsing microbubbles may be occurring 50.

Strategies deployed so far to control and sustain cavitation activity rely primarily on the pre-administration of ultrasound contrast agents, shelled microbubbles. The intended effect is mechanical in nature, resulting in bioeffects such as sonoporation or enhancement of vascular permeability. The latter mechanism is the subject of current clinical studies aiming at transiently opening the BBB for drug delivery 4,5. Monitoring and control of stable cavitation during ultrasound treatment have been reported in preclinical studies of BBB opening, as a reliable tool to ensure maintenance of safe and effective acoustic exposure level 51-53.

## 3. Applications of ultrasound-microbubbles antivascular treatments

Several applications of ultrasound-microbubbles induced antivascular effects. These include tumor growth control, radiosensitization, mechanical tissue lesioning, modulation of the immune micro-environment. These applications and associated mechanisms are summarized in Tables 3-5 and are discussed in detail in this section.

### Tumor growth control

Ultrasound-microbubbles induced vascular flow destruction has been reported to inhibit or delay tumor growth after treatment alone 45,47,54-56, or in combination with chemotherapy 47,48.

Observed tumor growth delays are thought to be the consequence of changes in blood flow, which include either a rapid permanent vascular shutdown or a temporary blood flow shutdown, following microbubbles destruction 45,47,57, as listed in Table 3. Acute complete shutdown of blood flow can occur within seconds after sonication 48 58. When sustained, these shut-down of blood flow are followed by widespread apoptosis and necrosis, see for example 45 and Table 3, consistent with ischemia, with the central regions of the tumor being preferentially affected by the exposures. This downstream necrosis of tumor cells is likely responsible for observed decrease in tumor growth, decrease in tumor volumes, and increased survival. Interestingly, even more significant tumor growth inhibition and improved survival was reported when another antiangiogenic medication (cyclophosphamide) was administered in concert with ultrasound- microbubbles induced vascular shut down 47.

Following continuous wave exposures, acute irreparable dilation of the tumor capillaries with associated intercellular oedema were reported, followed by the delayed liquefactive necrosis of neoplastic cells 59. The defective construction of tumor neo-vessels can account for their increased vulnerability to the effects of microbubbles destruction. A comparative study in immature and mature vessels revealed that while immature vessels were substantially depleted following microbubbles treatment, both in tumor and in muscle, the mature vessels resisted the effects of treatment in both tissues 55.

As the reduction of blood flow following insonification occurs rapidly, within seconds after ultrasound treatment, potential mechanisms most likely do not involve macrophage recruitment or changes in protein expression.

**Figure 3:**
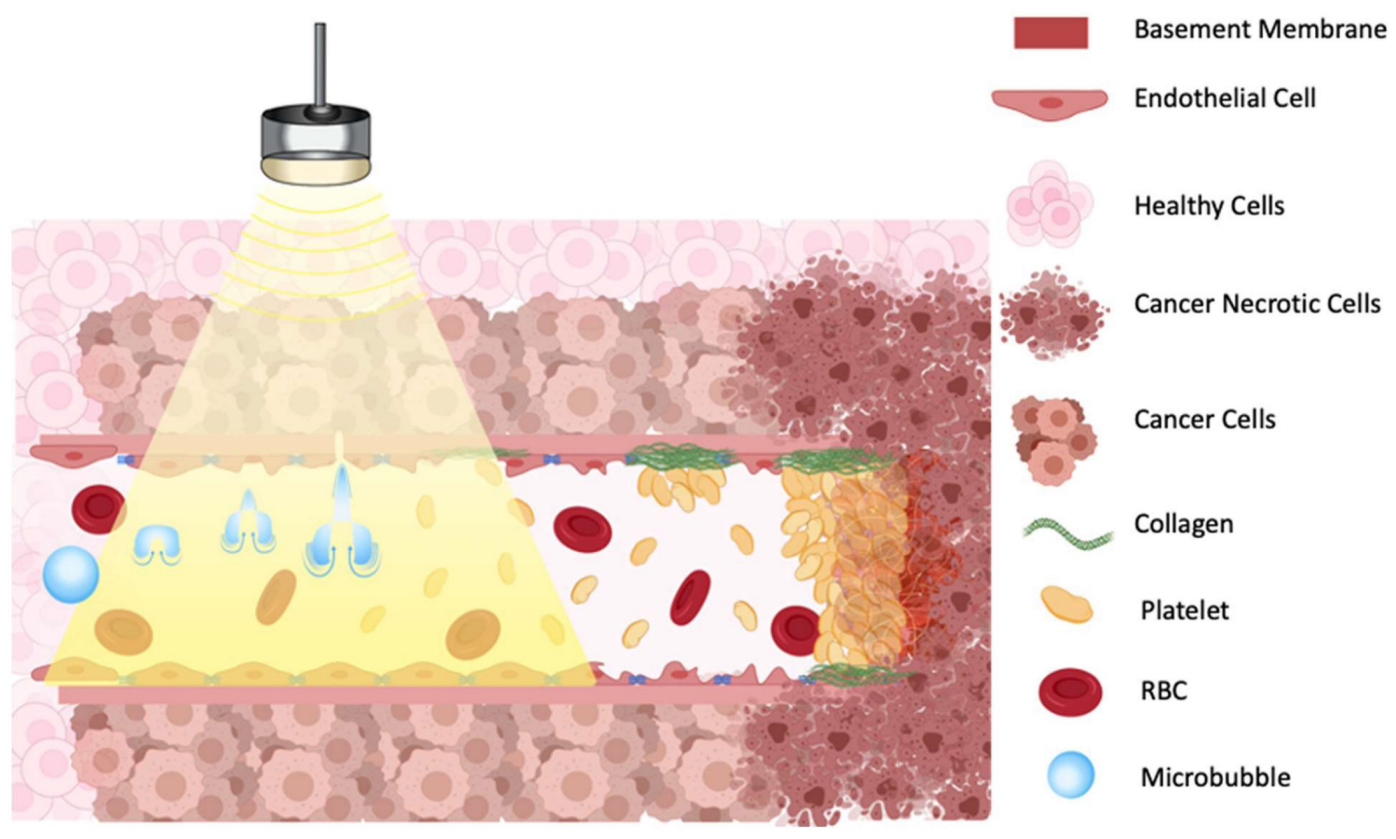
Illustration of proposed mechanism of action on how a combination of microbubbles and Focused Ultrasound damages endothelium, exposing basement membrane and causing aggregation of platelets, resulting in vascular shutdown.

One proposed model suggests that oscillating microbubbles mechanically damages the endothelium exposing components of the basement membrane including collagen 60, which then causes platelet aggregation and subsequent vessel thrombosis 61 (Figure 3). The platelet aggregation hypothesis was further validated by treating tumors with an anti-CD41 antibody, that binds on the surface of platelets and blocks potential thrombogenic effects, resulting in a greatly decreased reduction in blood flow after sonication 58. A comparative study in immature and mature vessels revealed that while immature vessels were substantially depleted following microbubbles treatment, both in tumor and in muscle, the mature vessels resisted the effects of treatment in both tissues 55.

There are multiple publications that confirm the link between direct damage to the endothelium and long-lasting vascular shutdown (Table 3). Severe damage to tumor vessels was reported to induce endothelial cells death resulting in vascular depletion and a decrease in tumor perfusion 55.

Damage to the endothelial cells however does not seem to be a prerequisite condition for blood flow shutdown after microbubbles treatment. Several studies reported temporary acute reduction of blood flow or blood flow reduction with blood flow restoration within 5 to 30 minutes after treatment 58,62 (Table 3). These transient reductions of blood flow were not accompanied by obvious structural changes or hemorrhage upon histology. This points to a biological amplification of the relatively limited contact between microbubbles and endothelial cells and suggests mechanisms such as vasospasm.

Several observations of the absence of evidence of coagulation necrosis immediately after microbubbles destruction, although extensive tumor cell necrosis and apoptosis were noted at later time points, suggest that blood flow disruption, and not heating, is the main mechanisms of action 55. Ultrasound induced thermal mechanisms, such as thermal ablation, are indeed associated with extensive coagulation necrosis 63,64.

Temperature effects cannot however be completely ruled out. Local temperature changes may occur following cavitation events and enhanced local energy absorption by microbubbles. Depending on treatment parameters, in particular the duty cycle (the fractional ON time), temperature increase due to insonification may be induced. Ultrasound treatment with high duty cycle is known to produce temperature elevation 65,66, while treatment with low duty cycles have not been associated with significant temperature increases (see Table 3). Temperature increases of up to 15°C was measured locally using continuous wave exposures for 3 min 67, of up to 5°C for 1% duty cycle, while no significant macroscopic thermal elevations were recorded at very low duty cycle (0.0001) 45. Heating arises from absorption of ultrasound energy by viscous damping of the oscillating microbubbles and is influenced not only by the properties of the microbubbles and the sonication parameters, but also by the blood flow, with slower flow leading to higher temperature increase 68.

### Radiosensitization

A second application of ultrasound-microbubbles induced vascular flow destruction is radiosensitization 69-74. Vascular shut down is a critical bioeffect by which radiation induces tumor cell death.

The ability of ultrasound-induced microbubbles destruction to act as a radiosensitizer holds great promise although the optimum protocol for combining two treatments is still unknown. Considerations should guide the timing of the sequence between microbubbles treatment and radiotherapy. One rational suggests that pretreatment with microbubbles can disrupt perfusion and damage tumor endothelial cells resulting in enhanced sensitivity to radiotherapy. Some studies reported optimized sensitization to radiotherapy when microbubbles treatment was performed a few hours before 70,71, whereas others have treated with radiation immediately after microbubbles treatment 75.

Ensuring complete vascular shut down is critical for radiation sensitization as radiation resistance may partly originate from tumor cell hypoxia, allowing cells to metastasize in the presence of reoxygenation. A recurring issue is that lower doses of radiation and other vascular disruptive agents may induce such hypoxia. Complete anoxia rather than hypoxia is desired as complete lack of oxygen results in rapid and complete cell death minimizing the ability for metastatic disease. Therefore, vascular shut down induced by ultrasound-induced microbubbles destruction is an exciting novel antitumor therapy.

The proposed mechanisms behind ultrasound-microbubbles radiosensitization relies on an evolving theory in radiation oncology that endothelial cell death with vascular shutdown is perhaps more important than the canonical radiation-induced DNA damage for tumor necrosis. This hypothesis is supported by studies where an inhibition of the radiation based vascular responses resulted in minimized treatment response 75,76. Radiosensitization following vascular shutdown triggered by ultrasound stimulated microbubbles was further enhanced when combined with a blocking antibody against delta-like ligand 4 (Dll4) 77, an angiogenesis deregulator. This triple combination led to a rapid tumor vascular-based collapse and a significant tumor growth delay.

**Figure 4:**
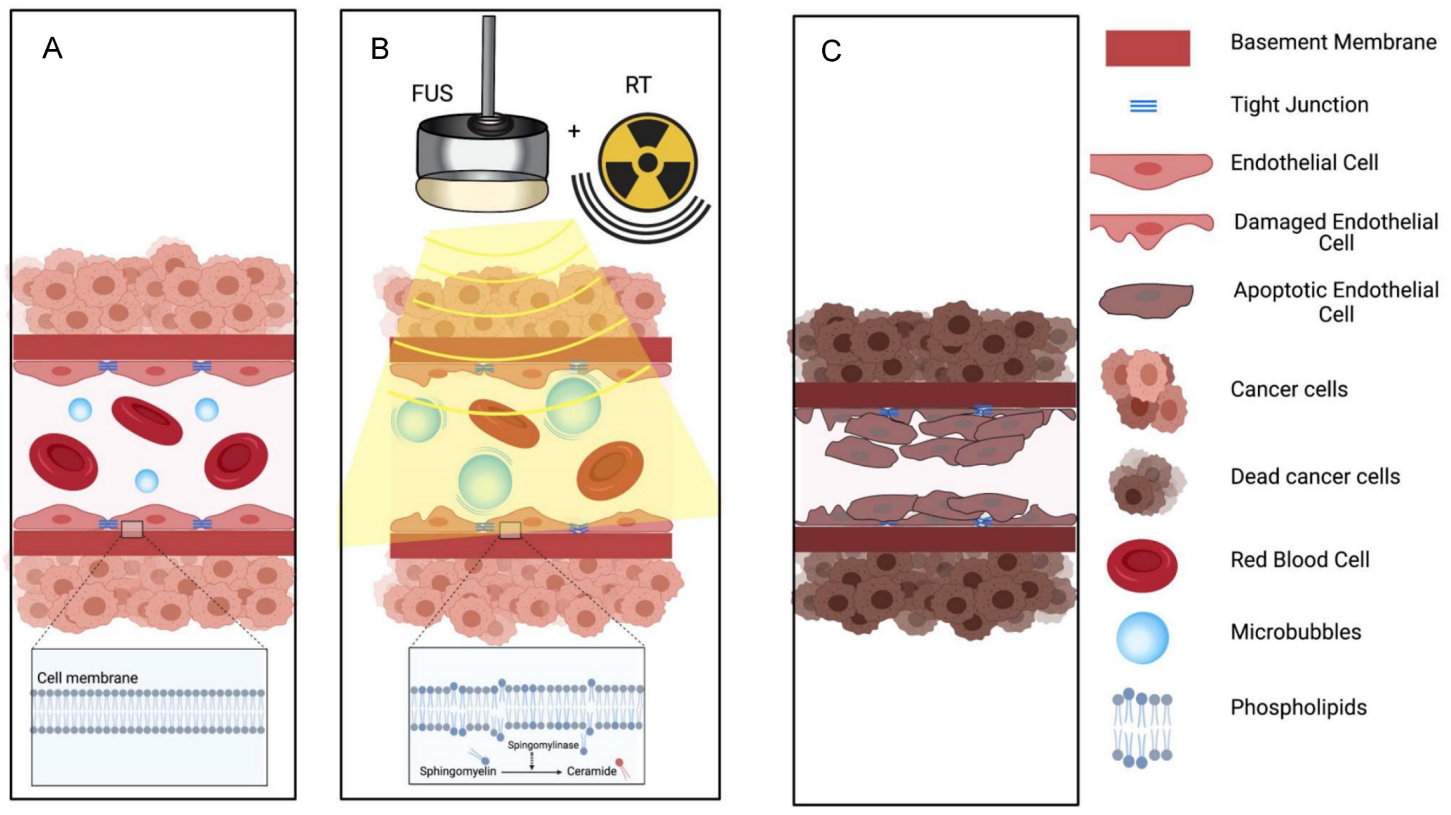
The ceramide model for vascular shutdown with endothelial cell death by ultrasound-induced microbubbles destruction combined with radio-sensitization. A: Injected MB circulates in the blood stream; B: MB destruction by FUS is combined with radiotherapy, causing in ceramide production, and C: resulting in endothelial cell death and subsequent vascular shutdown.

Ultrasound-stimulated microbubbles can cause endothelial cell damage and thrombosis with mechanisms similar to high dose radiation. High dose radiation has been shown to upregulate the amount of ASMase in the endothelium. This converts sphingomyelin into ceramide and induces endothelial cell death and rapid apoptosis. Decreasing the production on ASMase either by pretreating cells with basic fibroblast growth factor (bFGF) or sphingo-1-phopsphate (S1P), or by using ASMase deficient mice models resulted in the inhibition of ceramide-induced apoptosis 70. Endothelial cells also have 20x the amount of ASMase compared to other cells, which leads to a stronger response to radiation 78. Treatment with ultrasound and microbubbles can also increase the amount of ASMase 76 and ceramide 70,74,79 leading to endothelial cell death through perturbation of the cell membrane (Figure 4). Increased ceramide staining was demonstrated as early as 1 hour after microbubbles destruction *in vitro* 70; upregulation of genes that encode sphingomyelinase leads to cell membrane damage and de novo ceramide generation 80. Because of the known sensitivity of endothelial cell membranes to mechanical forces, the physical effects on endothelial cells induced by microbubbles destruction can interact synergistically with radiation therapy at relatively low (< 6 Gy) as well as at high (> 8 Gy) doses 69,70,73-77,80. Ultrasound alone or low dose radiation alone are not able to induce vascular shut down 79. Thus, the combination of ultrasound microbubbles-destruction and low dose radiation could be clinically beneficial, as it may provide an option to avoid high dose radiation, that leads to significant side effects.

Low-intensity pulsed ultrasound treatment, potentially inducing cavitation in the absence of microbubbles, was also shown to activate cell-surface receptors on the endoplasmic reticulum 81.

### Modulation of an inflammatory response

While a short-term response is associated with blood flow shutdown, at medium term, inflammatory response following capillary disruption has been reported to be associated with neo-revascularization, at least in muscle tissues, induced by brief microvascular remodeling response primarily manifested by changes in microvessels 82,83. The stimulation of expression of cytokines and adhesion molecules following cavitation events has also been used to enhance mesenchymal stem cell homing and engraftment 84,85, to treat myocardial infarction 86,87.

Even following non-inertial cavitation of microbubbles for BBB opening and drug delivery to the brain, acute transcriptional changes in the rat hippocampal microvessels following sonication are indicative of an initiation of angiogenic processes 88. A modest transient elevation of blood vessel density in the rat hippocampus 89 has been observed. When a high dose of microbubbles was used to open the BBB, using 10x a clinical dose Definity or 100µL/kg, an acute inflammation, with activation of the NFκB signaling pathway and immune activation, including Tnf, Birc3, Ccl2, accompanied by edema, neuronal degeneration, neutrophil infiltration, and microhemorrhage was also observed 90. BBB opening with microbubbles infusion of 100 μL of Optison was also reported to be able to induce sterile inflammation, with damage-associated molecular pattern response, concurrent with elevations in proinflammatory, 91 anti-inflammatory, and trophic factors, that lasted for 24 h 92. These treatments, performed with a high dose of microbubbles, typically 10x higher than the dose used for clinical imaging with Optison, may have resulted in an exaggerated inflammatory response, not observed when optimized parameters are employed.

### Lesioning of brain tissues

Initial attempts of using microbubbles destruction to induce brain lesions assumed that microbubbles would enhance the local energy absorption during focused ultrasound exposures 17,93-97. In fact, time-averaged acoustic power needed to produce localized necrotic lesions in the brain was less than one-tenth compared to the power required to produce thermal lesions without microbubbles in identical experimental conditions 98. It was also found that the peak temperature was likely not sufficient for thermal damage 99,100. These results suggest that the low-level heating produced during cavitation was indirectly related to the production of the target lesion at this set of ultrasound parameters. Further studies by the same group demonstrated the formation of lesions in the brain relying on mechanical effects of microbubbles-induced cavitation, without heating 98,101 (Table 4).

When microbubble-induced cavitation is applied to induce lesions in normal brain tissue (Table 4), these lesions are formed presumably via mechanical vascular damage and subsequent ischemia in downstream tissues and localized ischemic necrosis. Lesions are formed with a central region containing red blood cell extravasations surrounded by edema 49. In the case of brain tissue, however, it is not clear that the mechanically induced damage to the vasculature results in an immediate blood flow shut down. Lesions have been produced while T1w MRI imaging suggested no stop in blood flow just after the treatment 102, while microhemorrhages and lesion formation timeline were consistent with tissue necrosis after ischemic stroke 101. Histologic findings suggest FUS with microbubbles in the brain might induce two cavitation-mediated processes, ischemia resulting from occlusion of the capillary blood vessels (through the formation of emboli and platelet aggregation) and inducing localized lesions dominated by apoptosis 91, but also potentially some hemorrhagic necrosis 103. Importantly, animals followed over 9 weeks showed no delayed hemorrhages 101.

### Enhancement of response to chemotherapy

Another application of microbubbles destruction is to enhance the effects of chemotherapy by acting as a vascular disruptive agent to destroy the fragile central blood vessels within the tumor and allow for increased delivery of chemotherapy to the tumor periphery 48. Alternatively, microbubbles destruction can be used to thrombose vessels after targeted drug delivery to enhance drug retention by decreasing washout. microbubbles destruction proved efficacious when coupled with drugs, enhancing anti-tumor effects such as metronomic cyclophosphamide 47 or low-dose docetaxel 48. Coupling of microbubbles destruction with liposomal doxorubicin (Doxil) had also been proposed, under the rationale that vessel damage and reduction in tumor perfusion resulting from microbubbles destruction would improve intra-tumoral drug uptake 104. Thus, the effects of vascular shut down to enhance the effects of chemotherapy is likely multifactorial, from destroying intrinsic tumor vessels causing necrosis, to enhancing the efficacy of targeted drug delivery, while also limiting the supply of essential nutrients and oxygen to cells. This approach, involving relatively low acoustic pressures compared to ablation techniques, could be well suited for the treatment of locally advanced tumors, for example in a neoadjuvant context, and could facilitate the treatment of tumors in the brain, kidneys and liver, where acoustic focusing and/or tissue motion can present challenges for ablative therapy.

### Modulation of tumoral immune micro-environnement

Another proposed mode of action of vascular shutdown for antitumor therapy is to trigger an antitumor immune response following endothelial damages. Most preclinical and clinical literature suggests that ultrasound microbubbles destruction may trigger anti-tumor immunity by induction of specific inflammation, modulating immunosuppressive cytokine expression, releasing endogenous danger signals such as heat shock proteins and tumor antigens which can stimulate leukocyte infiltration and activation mostly through increased vascular permeability 105-107. When low intensity microbubbles destruction was applied to a subcutaneous model of melanoma, there was increased infiltration of HIF1A+ (hypoxia inducible factor 1A+) cells indicative of necrosis and increased CD45+/CD3+ T-cells into the tumors 108. Similarly, in a subcutaneous mice model of colon carcinoma, non-T regulatory tumor infiltrating lymphocytes and continual infiltration of CD8+ cytotoxic T-lymphocytes with increased CD8+/Treg ratio was reported with low pressure pulsed ultrasound destruction of microbubbles via increased permeability of the vasculature without hyperthermia, and microbubbles destruction had increased antitumor immune response with decreased tumor growth compared to no changes without microbubbles. 109.However, a more recent study did not observe a shift in T-cell population into a more immunocompetent state when microbubbles destruction was combined with immunotherapy in the later subcutaneous model of colon carcinoma 110. Such apparent contradictory results may be the consequence of utilizing different US treatment parameters. These studies, indeed, used different treatment settings, pulsed sonications for 110 and continuous sonication for 108, reported to possibly induce hyperthermia 67 at different pressure amplitudes. These elicit different bioeffects, with one treatment resulting in no apparent impact on perfusion but increased vessel permeability and erythrocyte extravasations 109, while the two others resulted in shutdown of blood flow accompanied by necrosis 108,110.

Moreover, analyses of the immune infiltrate were conducted at different time points in these different studies, and the dynamic aspect of an immune-modulation following ultrasound-microbubbles treatment is yet unknown. These reports may also suggest that the exact exposure conditions and associated parameters are of utmost importance in the resulting immunomodulation, as has also been observed with ablative therapeutic ultrasound modalities 105.

Antitumor immune responses have also been noted with non-ablative ultrasound treatment. In the absence of microbubbles, the treatment of a primary B16 mouse model of melanoma with mechanical focused ultrasound utilizing sufficiently high peak pressure to presumably induce cavitation, was shown to reverse T cell anergy and activate dendritic cells in the tumor and tumor-draining lymph node, with the translocation of surface calreticulin and activation of heat shock proteins (HSPs) which were mechanisms to trigger this immune response 111. Additionally, treatments with pulsed ultrasound at peak negative pressure of 4 or 6MPa, also likely to be mediated by cavitation, have been reported to modulate an anti-tumor immune response in murine melanoma (B16) and breast (4T1) tumor models, in particular through suppression of anti-inflammatory cytokines 112. Whether or not similar mechanisms can be activated following microbubbles destruction remains to be studied.

## 4. Role of treatment parameters

Anti-vascular effects have been reported following a very wide range of exposure conditions, including continuous wave exposure, very short duty cycles, low and high peak negative pressure 43,45,52,53. The proposed models and mechanisms of action discussed above are summarized in the Table 2. These involve damage to the endothelium, thermal effects and mechanical lesioning. These effects are governed by microbubble dynamics, which in turn will vary depending on several factors including the ultrasound pressure treatment parameters, the microbubble population characteristics, local microenvironment, and tissue type. The role of these treatment parameters is discussed in detail in this section.

### Exposure conditions

Antivascular effects have been reported following a very wide range of exposure conditions. Effects have been achieved following continuous wave exposure at low peak negative pressure (or PNP) of about 0.25 MPa 54,67 or very short duty cycles, down to 0.0001 at high PNP (> 1.5MPa). When a range of pulsed-US conditions was examined, it was found possible to induce flow inhibition effects, along with necrosis and apoptosis, using relatively low duty cycles (fractional ''on-time'' of US; 0.0001-0.01), though the degree of these effects decreased with duty cycle 45. Vascular shutdown was also reported using very low duty cycle (0.00024) 47.

Two scenarios can therefore be proposed. In the first one, relatively low peak negative pressure is applied, leading to predominantly stable non-inertial cavitation and a dynamic of microbubbles volumetric changes dictated by the ultrasound field. In the second scenario, higher peak negative pressures are applied leading to transient inertial cavitation and bubbles collapse and fragmentation 37,38. Stable non-inertial cavitation has been associated with transient bioeffects such as increased vascular permeability without endothelial cell damage, necrosis or erythrocytes extravasation 113, although these effects will depend on the acoustic pressure and frequency 60. Blood-flow or vascular shutdown have been associated with the onset of inertial cavitation 22. Therefore, the pulsing sequence becomes a primordial parameter to initiate and control the cavitation activity, based on the probability of occurrence and spatial distribution of cavitation, to induce the desired bioeffects.

Ultrasound treatment sequence, defined by its center frequency, peak rarefractional pressure, the pulse length, the repetition frequency of the pulse and the total insonification time, will impact microbubbles dynamic. The ultrasound pulse shape, and especially the peak negative pressure will dictate the microbubbles dynamics on a microsecond scale, with higher pressure levels leading to a shift from stable non-inertial to transient inertial cavitation 38. However, it is the pulse sequence that will dictate the number of times these dynamics occur and the location where they occur 41. The shape and sequence of ultrasound pulse have been demonstrated to control magnitudes, types and durations of cavitation events within the focal volume, and a heterogenous distribution of cavitation activity can lead to a heterogeneous distribution of bioeffects 114. This is especially relevant with long pulses that will result in an upstream destruction of microbubbles flowing into the ultrasound field, inducing an upstream accumulation of cavitation events.

To control the spatiotemporal distribution of acoustic cavitation activity, novel sequences, named rapid short-pulse (RaSP) sequences, based on short pulse sonication (5 to 50 cycles) with short (microsecond order) off-times, were proposed 115. These RaSP sequences have been shown to improve the lifetime of flowing microbubbles and the spatial homogeneity of cavitation *in vitro* when compared to long pulses of 50000 cycles 115. When applied to BBB opening in preclinical animal studies, these sequences provided uniform drug distribution in the brain parenchyma 41,116, with optimal BBB opening achieved for series of very short pulses at high PRF (100kHz) repeated at 5Hz 38 or 1.25kHz repeated at 0.5Hz 116. These RaSP sequences may however require higher dose of microbubbles to be as efficient as longer tone burst sonication to open the BBB 117. These parametric studies on the influence of ultrasound sequence on the BBB opening may bring very useful lessons for the design of future microbubbles destruction sequences aiming at blood flow or vascular shutdown.

The limit of this comparison however lies in the low PNP used for BBB opening, typically lower than 1MPa 113, at the edge or lower than the inertial cavitation threshold. Whereas the use of short pulses can lead to a higher spatial extent of cavitation events (because of an increased lifetime of flowing microbubbles) - remains to be investigated for PNP of several MPa typically used to induce vascular damages. When violent inertial cavitation is likely to take place, the questions of microbubbles persistence or lifetime, and of the replenishment of the vasculature before the arrival of subsequent acoustic pulses, remain central to the design of ultrasound sequences.

If ultrasound acoustic pressure is sufficiently high for the destruction of microbubbles, PRF may come into play, in relation to blood flow rate in the microvasculature. If acoustic pulse repetition is fast, microbubbles may be destroyed in larger vessels, well before reaching the capillaries, because blood flow in those vessels is relatively slow. If ultrasound pulses are intermittent (e.g., frame rate of 1 Hz is quite common), microbubbles that travel from larger vessels through arterioles may reach the capillaries, before they are destroyed in the bulk blood. At that point, microbubble insonation will lead to bubble expansion to touch endothelium that surrounds the bubble in the capillary. Vessel damage has been reported, even to the point of rupture, RBC leakage, and petechial hemorrhage formation 118.

In the presence of microbubbles, the bio-effects have been achieved with pressure amplitudes lower than would be used in ablative HIFU. Ultrasound exposure slightly more powerful than diagnostic levels, yet significantly lower than the range used for thermal and ablative treatments, can cause a temporary disruption of tumor blood flow, which can last about ten minutes 62. The peak pressure however, needs to be above a threshold for microbubbles destruction to occur 119, which will depend on the frequency, the pulse duration, the PRF, the microbubbles type and concentration. When using microbubbles targeting the vascular endothelium, reducing the peak negative pressure decreased the occurrence of regions with reduced blood flow 58. Studies comparing anti-vascular effects at different pressure amplitude did not report a significant reduction in mean vascular density after treatment at 0.5MPa at 1MHz 55, and no visible damages were observed on the endothelium for pressure below 0.85MPa at 2.25 MHz 46. Most of the anti-vascular effects by microbubbles destruction were obtained with pressure typically above 1MPa. Although inertial cavitation from ultrasound contrast agents have been reported for peak negative pressure typically starting at 0.5MPa, higher levels of pressure are required to ensure that inertial cavitation is triggered in a significant population of microbubbles, at least for short pulses duration 119. Studies reporting reduction of tumor perfusion at lower pressure were likely to have been dominated by thermal effects 67.

### Microbubbles injection route

Combination of route of administration and selected exposure parameters, especially the PRF, may also be an important factor to induce bioeffects. Comparison of studies performed with similar low duty cycles, show that some induced significant tumor growth delays 47 while some did not 45. One of the major differences here was the manner of injection, with one study using a large (20s) inter-burst interval to allow microbubbles reperfusion between pulses 47, and the second using shorter inter-burst interval (5s) 45, possibly not long enough to allow replenishment. While no definitive conclusion can be drawn from this comparison because of differences in tumor model and the treatment schedule between these two studies, it can be anticipated that the use of a longer inter-pulse interval should allow replenishment and result in more sustained effects.

When a bolus injection is used, the concentration of microbubbles will vary over time, as evidenced by the dynamic enhancement with contrast-enhanced ultrasound (CEUS) imaging 120. These variations suggest that over a long treatment period, such as 3min 48 or 60min 45, the induced bioeffects will decrease over time, if not disappear, and may require multiple microbubbles injections to be sustained. Variation of microbubbles concentration over time also requires a great control over the timing between the microbubbles injection. When microbubbles are injected in bolus, the sonications should typically start a few seconds (15s in 48) after the start of the injection in order for the microbubbles concentration to reach its peak levels in the region of interest.

In order to optimize treatment efficacy, it is required to ensure that microbubbles are still circulating at the time of the sonication. This can be achieved with an a priori knowledge of microbubbles circulation time and/or real time CEUS imaging. The contrast reperfusion time has been used to determine an optimized timing between burst, such as 20s in 48. Cavitation monitoring can also be a way to quantify the induced physical effect produced by the insonification of the microbubbles.

### Microbubbles dosing

A recent study examined the influence of microbubble parameters, such as size and concentration on BBB disruption. Rats were treated with cationic microbubbles (Advanced Microbubble Laboratories, Boulder, CO, USA composed of DSPC/DSTAP/DSPE-PEG-2K) 121, and sonicated for 5 min (1MHz, 1 MPa PNP, PRF 100Hz, 10%DC) 121. Using either 2 or 6µm diameter microbubbles, and gas volume doses varying from 1 to 40 µL/kg, it was determined that total gas volume in the administered dose, and not the microbubbles diameter, was the main factor determining the extent of BBB opening, with a linear increase for both diameters.

Whether microbubbles volume could be a unifying parameter in anti-vascular microbubbles ultrasound remains to be investigated. There is also a relationship between microbubbles size and concentration and the persistence in circulation. It has been reported that for matched concentrations, larger microbubbles were more persistent in circulation, but when volume matched, all microbubble sizes had a similar circulation half-life, as evidenced by high-frequency contrast imaging in mice kidney 122.

**Figure 5:**
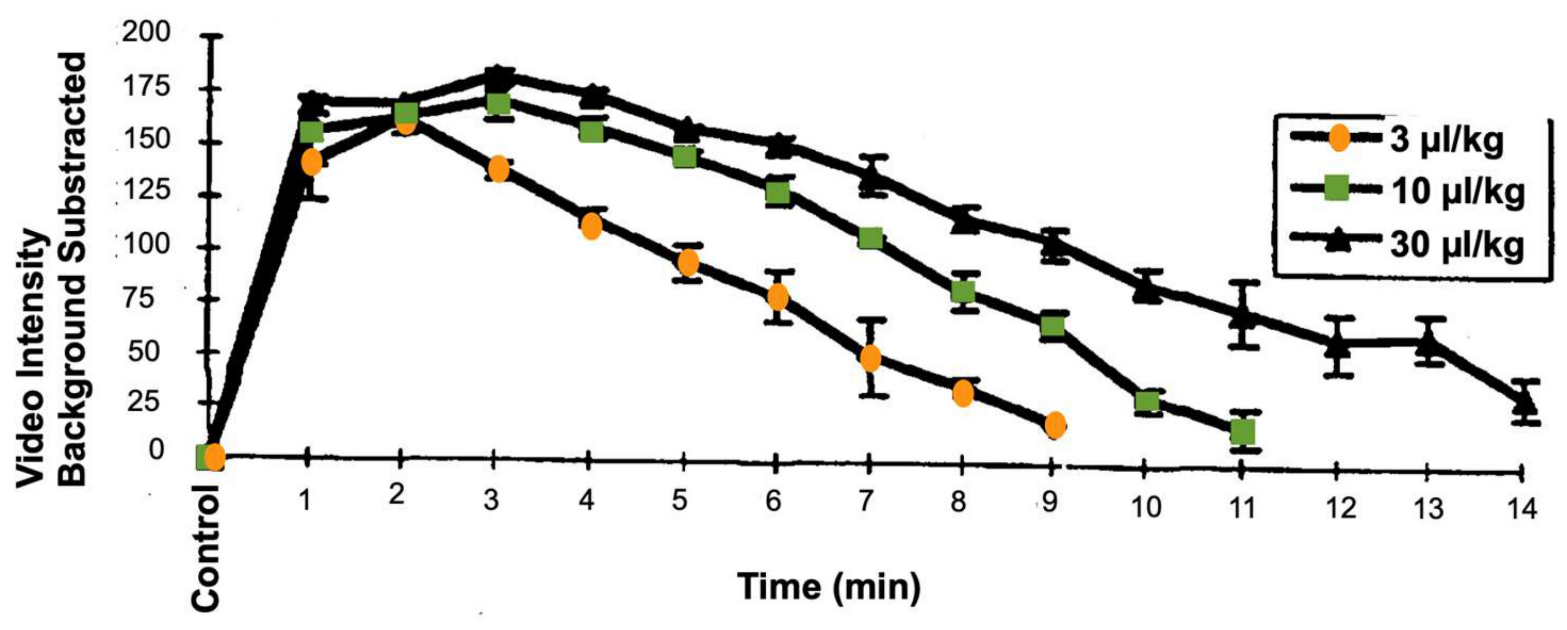
Time-intensity curve for left ventricular opacification and second harmonic imaging in anesthetized dogs, after infusion of Definity microbubbles at different concentration. Each line is the mean +/- SEM (Standard Error of the Mean); N=4; EOI = End of infusion. These data are publicly available on the FDA website, as part of the report “Review and Evaluation of Pharmacology/Toxicology Data” for NDA number 21-064, Definity microbubbles.

Generally, microbubbles signal in an intraoperative scan is observed starting from 20-30 seconds after an intravenous bolus injection. In the brain, for example, a complete wash-out depends on the distribution within different areas and tissue types 123,124. Intravascular lifetime of second-generation ultrasound contrast agents, with a poorly soluble fluorinated gas core, allows the study of structure/organ for several minutes 125. Circulation times of the clinically approved microbubbles (Sonovue/Lumason, Optison and Definity) have been described in the literature, in multiple preclinical animal tests and in clinical trial reports of ultrasound contrast imaging blood pool enhancement. They tend to be relatively similar, several minutes following bolus injection or short infusion 126,127. Contrast enhancement time is prolonged upon the increase of the administered dose (Figure 5). Circulation of second-generation microbubbles agents can be further improved with novel formulations. The BR38 microbubbles from Bracco for example, which incorporates a mixture of perfluorobutane and nitrogen gases, has a body elimination half-life of 10 minutes in humans 128. Polymer microbubbles such as PBCA (poly(n-butylcyanoacrylate)) may also provide prolonged circulation time 30.If prolonged insonation treatment is required, bolus administration of microbubbles may be substituted with continuous infusion, which is also an approved technique for contrast ultrasound imaging (see e.g., package insert for Definity or Lumason).

There is an ongoing debate regarding the optimal microbubbles dose, especially for the brain treatment applications. A significant and damaging inflammatory response was observed at high microbubbles doses in preclinical models 90. But it was also demonstrated that ultrasound with reduced microbubbles dose can induce increased BBB permeability without an associated upregulation of NFκB signaling pathway gene expression 90. This emphasizes the importance of employing optimized ultrasound parameters and microbubbles dose to mitigate the chances of causing injury to the brain at the targeted locations.

Since there is no regulatory approval for the therapeutic use of microbubbles, we analyzed the preclinical studies based on the FDA-approved doses for diagnostic uses and compared it to a “clinical” dose of microbubbles. To induce downstream ischemic lesions, the amount of microbubbles used, normalized in terms of number of microbubbles/animal weight, ranged from 10^8^ MB/kg when microbubbles are administered in an infusion mode 45 (20x the clinical dose of Definity), up to 10^10^ MB/kg 110 or 10^11^ 108 in bolus for soft tissue applications, which, depending on what microbubbles are used, can represent up to several dozen times the clinical diagnostic dose for a single bolus. For nonthermal ablation of normal brain tissue, localized ischemic necrosis could be achieved with much lower dose, 2- to 6-fold higher than the Optison clinical dose 101. Postulated mechanisms of the observed bioeffects of microbubble insonation in the vasculature imply that the bubble should be located in close proximity to endothelial lining. Most efficient scenario for the induction of detectable bioeffects implies expansion and compression of microbubbles during insonification cycles, at the time when those bubbles are located within the narrow confines of blood capillaries. In that case, expansion of the bubble during rarefaction will lead to close contact of bubble surface with endothelium, which sometimes leads to deformation of the capillary wall 129. Obviously, the probability for the bubbles to induce bioeffects will increase with the injected dose of the particles.

Studies using ultrasound-induced microbubbles destruction to induce microvascular damages for radiosensitization used doses much higher than approved for diagnostic imaging purposes. The range was from 2.10^8^ MB/kg of Optison (exceeding 55 clinical dose by 22-fold) 71, up to 10^9^ MB/kg of Definity, (10- to 300-fold the clinical dose) 70. Although most studies used high microbubbles doses, treatment parameters optimization is possible to achieve enhanced radiation response with clinically used microbubble concentrations combined with ultrasound 130.

These comparisons are difficult because of variations in microbubbles size and compositions (shell and gas), and by other influential ultrasound treatment parameters (frequency, pressure amplitude, DC, PRF, repeated or single treatment) or even the acoustic field (plane v. focused transducers, pressure amplitude). It is notable that achieving microvascular damage through such a wide range of microbubbles concentration can be achieved.

## 6. Discussion

This review summarizes the current knowledge of ultrasound-induced microbubble destruction to induce antivascular effects. Complete vascular shutdown is the result of cavitation induced microbubbles damage to the endothelial cells either causing platelet aggregation to the exposed basement membranes or via the mechanical activation of the ceramide pathway inducing endothelial cell death and apoptosis. A variety of desired bioeffects can result from antivascular treatment with cavitation alone such as ischemia with tumor necrosis and nonthermal brain lesioning, while in combination with other strategies this treatment can increase radiation sensitization, enhance the effects of chemotherapy, and possibly elicit an anti-tumor immune response. As described in this paper and presented in Tables 3-5, there is significant variability in the ultrasound treatment parameters and the microbubbles administration parameters.

### Microbubbles dose and injection routes

In order to safely and effectively induce antivascular effects from cavitation treatments, careful integration of technical focused ultrasound beam parameters and details of microbubbles administration should be considered. For microbubbles, such details include administration mode (intravenous bolus or infusion), time interval between sonications, and microbubbles concentration, size, and composition. The transducer parameters and treatment approach may also be varied, by using manual or electronic steering, quantifying the amount of tumor coverage, and defining pressure settings. In the case of BBB opening, where only transient bioeffects are needed and inertial cavitation is excluded for safety concerns, two different regimens are utilized with either a fixed pressure 4, or an optimization of pressure calculated as 50% of the power at which cavitation signals are first detected using acoustic feedback from an incremental sonication power protocol 52,131. For other applications, different pressure levels have been used, but in order to induce microbubbles cavitation, pressure levels were typically above 1MPa for frequencies around 1MHz (Tables 3, 4 and 5) for microvascular ablation, brain tissue lesioning or radiosensitization, or above 0.5MPa for lower frequencies (500kHz); in any case to be above the microbubble's cavitation threshold.

Based on the microbubbles injection mode, timing of ultrasound bursts must be carefully selected to allow reperfusion by microbubbles between two successive pulses. These treatment parameters should also be optimized for specific locations, as microbubbles spatial distribution may greatly vary between organs, and between structures within a given organ such as brain. Pilot studies should be conducted to examine contrast reperfusion time, and a lot can also be learned from ultrasound imaging of microbubbles. In the brain for example, the spatial and temporal distribution of microbubbles is still an area of open investigation, but microbubbles imaging has already provided valuable information. Intraoperative microbubbles contrast-enhancing patterns of intracranial neoplasms have been qualitatively characterized 123,124, with significant differences reported between various subtypes. For example, glioblastoma demonstrated a highly heterogenous and intense pattern of microbubbles, with clearly identifiable arterial suppliers and much faster arterial phase and enhancement peak when compared to lower grade gliomas, which have weaker, more homogenous and slower transit with delayed venous phases. Arterial suppliers and draining vessels are also less clearly visible. Thus, intraoperative differential diagnosis of tumor subtypes (not limited to gliomas) might be achievable through CEUS. Similarly, differences in microbubbles contrast enhancement patterns have been observed between different structures, such as white matter, grey matter, basal ganglia and vascular structures. However, a more standardized, reproducible and quantitative approach would surely be desirable when addressing therapeutic approaches rather than real-time imaging. In this regard, a recent work showed that using time intensity curves for accurate quantitative analysis of microbubbles distribution in CEUS intraoperative imaging enables differentiation of brain tumors as well as variations of flow in different areas of the brain 132. Such a quantitative approach, once the relation between microbubbles concentration and effects of FUS is established, may allow in the future to optimize treatment outcomes using patient-tailored treatment for maximum therapeutic impact to targets while sparing unaffected areas nearby.

Determining the optimal microbubbles dose in combination with ultrasound beam parameters is critical to mitigate injury at the targeted location. Since there is no regulatory approval for the therapeutic use of microbubbles, we analyzed the preclinical studies based on the FDA-approved clinical doses of microbubble contrast agents, as prescribed for diagnostic ultrasound imaging. Doses of microbubbles differ greatly between published microbubbles-based anti-vascular studies, as summarized in Tables 3-5 but in general are significantly higher than the amount approved for diagnostic imaging. The amount of microbubbles used, normalized in terms of number of MB/animal weight, ranged from 10^8^ MB/kg for infusion 45 which is approximately 20 times the clinical dose of Definity, up to 10^10^ MB/kg 110 or 10^11^MB/kg 108 as a bolus for soft tissue applications, which can represent more than ten times the clinical dose for a single bolus. For brain applications, localized ischemic necrosis lesioning could be achieved with a much lower dose, only 2 to 6 times the Optison clinical diagnostic dose 101.

When using ultrasound stimulation of microbubbles for BBB opening, a high microbubbles dose using 10x the clinical dose Definity or 100µL/kg, elicited acute inflammation with activation of the NFκB signaling pathway and immune activation, accompanied by edema, neuronal degeneration, neutrophil infiltration, and microhemorrhage^90^. However, safe BBB opening can also be performed without an associated upregulation of NFκB signaling pathway gene expression by changing protocol settings 90.

Finally, studies using a wide variation of microbubbles doses can induce microvascular damages for radiosensitization ranging from 2.10^8^ MB/kg of Optison, or 22 times the clinical dose 71, up to 10^9^ MB/kg of Definity, 10 to 300 times the clinical dose 70. These comparisons are difficult to interpret because of variations in microbubbles size, composition (shell and gas), and other technical factors in the US beam properties.

### Timing of ultrasound microbubbles destruction treatment with combination therapies

If drugs are used in association with microbubbles treatment, their pharmacokinetic properties are a crucial factor to determine the timing of their injection. Injection of drugs before microbubbles allows enhanced drug penetration within the tumors. Time interval between drug injection and microbubbles treatment must be carefully selected with respect to the drug plasma half-time, to avoid situations where microbubbles treatment would start too late, after the drug has already been cleared from plasma.

Same considerations should guide the timing of the sequence between microbubbles treatment and radiotherapy when used in association. One rational for this association is that pretreatment with microbubbles can disrupt perfusion and damage endothelial cells in tumors, resulting in enhanced sensitivity to radiotherapy. The time interval between microbubbles treatment and radiotherapy should take into consideration the dynamics of perfusion shutdown. This time interval is not yet standardized as some studies reported optimal sensitization to radiotherapy with microbubbles administration a few hours before 70,71 or immediately after microbubbles treatment 75. A recent study evaluating multiple time intervals with ultrasound microbubbles destruction before and after radiation, suggested vascular normalization as a consequence of a combinatorial therapy 133.

### Novel applications of intravascular ultrasound-microbubble treatments

Novel applications of microbubbles-microvascular bioeffects that induce vascular shut down are being proposed. Simulating the spatial resolution of a laser to precisely nucleate bubbles in selected vessels, synchronized with an acoustic pulse (1 MHz at 0.45 MPa and 10 Hz with 10% duty cycle), was recently reported to induce an almost 70% reduction in blood perfusion after 7 days in a rabbit ear model, without damaging the surrounding cells 134. The combination of tumor microvascular injuries and embolization with sonodynamic therapy using berberine (BBR) nanoparticles as a sonosensitizer demonstrated suppression of tumor proliferation 135. Another use of microbubbles-induced cavitation has also been studied to improve atherosclerotic plaque stability by selectively destroying the intraplaque neovasculature with a treatment at 3MPa through a reduction in erythrocyte extravasation and inflammatory mediator influx 136. Destruction of microbubbles was also used to deliver IL-8 monoclonal antibodies in a rabbit model of atherosclerotic plaques and was shown to alleviate inflammation 137.

Interestingly, microbubbles destruction is now investigated as an adjunct to microwave ablation. The significantly reduced tumor blood perfusion can lead to a sharp rise in the local temperature of the treatment area, resulting in increased necrosis and apoptosis in a murine model of hepatocellular carcinoma (HCC) 138.

Other potential clinical use of microbubbles focused ultrasound is to enhance vascular permeability and cell transfection in the brain. For example, significant preclinical studies have demonstrated the ability of low intensity focused ultrasound to enable liquid biopsy in the brain by opening the blood brain barrier. Following treatment or brain tumors in mice with pulsed ultrasound and microbubbles (1.5MHz, 1.5MPa, pulse length 10 ms, PRF pulse, for 30 s at 4 different locations), an increase of up to 4800-fold in a reporter mRNA (eGFP) was found in the peripheral blood samples compared to untreated mice 139. Follow-up studies in small and large animals with MRI imaged guided brain-tumor biomarker release confirmed the efficacy and the safety of the technique 140,141. A proof-of concept study reported the ability of MRgFUS to enhance the release of circulating brain-derived biomarkers including cfDNA, extracellular vesicles, and brain specific protein, demonstrating the potential of the technology to support liquid biopsy for the brain 142.

Activation of cationic microbubbles coated with plasmid DNA microbubbles with very low-pressure focused ultrasound (0.1 MPa, 1.1 MHz) selectively and effectively delivered genetic material to the targeted cerebral microvessels in mouse brain, without disrupting blood-brain barrier integrity or eliciting detectable inflammation, opening novel perspectives in sono-selective transfection 143.

The destruction of microbubbles at the reduced acoustic power levels with magnetic resonance imaging (MRI)-guided focused ultrasound (MRgFUS) was reported as a tool to achieve mild hyperthermia. When combined with liposomal doxorubicin (Caelyx), this hyperthermia resulted in increased levels of drug in muscle but not tumor tissue, suggesting a complex interplay between the heating effects of microbubbles with those of enhanced permeabilization and possible vascular damage in the tumor 144.

An interesting example of microbubbles-mediated bioeffects outside the brain is related to sonoporation and increased vascular permeability for enhanced drug delivery. It has been applied in an early-stage clinical trial with encouraging results to deliver gemcitabine in pancreatic tumor of inoperable patients 145, and there is a plethora of preclinical data 8,146.

Perhaps one method of improving the ability to control cavitation induced vascular shut down involves exploring the use of other agents that can also interact with ultrasound to induce cavitation-related bioeffects. Acoustically activated droplets, also known as phase-change emulsion 147 droplets, can vaporize under ultrasound stimulation 148. They can be potentially used with spatial and temporal control resulting in targeted tissue occlusion 149,150. Novel agents, such as an ultrasound-responsive single-cavity polymeric nanoparticle, has been recently shown to actively transport and improve the distribution of therapeutic agents in tumors 149,151, and can enable the extensive extravasation at low acoustic energies 152. To our knowledge, these agents have not yet been investigated for anti-vascular therapy. They can present some potential advantages over microbubbles, such as longer circulation time, and capacity to extravasate.

### Imaging for treatment planning and monitoring

All these treatments are intrinsically linked to imaging for treatment planning, targeting, monitoring and follow-up. Ultrasound imaging seems the most natural imaging modality, as it can monitor blood tissue perfusion and blood velocity in the vessels, especially when enhanced by microbubble contrast for imaging or for microbubble-enhanced Doppler 153. Microbubbles contrast imaging is for example used to assess tissue destruction following thermal treatment with high intensity focused ultrasound (HIFU) of prostate cancer 154,155. This imaging is performed while the patient is still in the operating room, so immediate re-treatment is possible in case of an unsatisfactory result, demonstrating that the unwanted bioeffects of microbubbles in a therapeutic ultrasound setting can also be safely mitigated.

More recently developed modalities of ultrasound imaging have open prospects for a detailed characterization of vessel morphology and blood flow characterization. Two proposed methodologies have pushed the limits of what is achievable in terms of imaging resolution of blood vessels with ultrasound. The first one, named acoustic angiography, is a dual-frequency contrast-enhanced technique, using a regular emission frequency (a few MHz) for excitation of microbubbles, and a very high receiving frequency (typically between 10 and 20 MHz) for constructing images with a better resolution than otherwise achievable with a single transmit-emit frequency. When implemented with dedicated transducers and image processing, this method can generate images with a resolution down to a few tens of microns 156-158. The second approach, named super-resolution imaging, relies on specific ultrafast acquisition techniques and post-processing, to image at resolutions beyond the wavelength limits of the device. Several postprocessing algorithms for CEUS image analysis have recently been proposed for a detailed characterization and quantification of vascular features at super-resolution 159-161, and could also open novel ways to plan and monitor treatment response.

### Clinical translation

With the knowledge gleaned from the preclinical work with ultrasound vascular ablation, researchers at Thomas Jefferson University recently published the results of the first pilot clinical study using ultrasound-triggered microbubbles destruction for augmenting hepatocellular carcinoma response to transarterial radioembolization (NCT 03199274) 29. There was a greater prevalence of tumor response in participants who received a combination treatment, i.e., ultrasound-triggered microbubbles destruction and radioembolization versus radioembolization alone, with no significant complications. The patients receiving both treatments received 3 separate microbubbles destruction sessions 1-4 hours and approximately 1 and 2 weeks after radioembolization. An ultrasound triggered microbubbles destruction sequence was used with a mechanical index of 1.13 at 1.5MHz transmitting at 2.3 µsec pulses and a PRF of 100Hz and repeated for several minutes 27. Two other clinical trials that use ultrasound microbubbles destruction for radiosensitization for head and neck cancers (NCT04431648) and chest-wall/locally-advanced breast cancers (NCT04431674) are currently recruiting patients.

## 7. Conclusion

Ultrasound-induced microbubbles destruction antivascular effects can potentially be utilized as a focal, targeted, noninvasive treatment, alone or in combination with other treatments. Additional knowledge gaps should be investigated to allow further advancements and allow for increased translational research. It is likely that this treatment modality will bring more clinical trials, when the questions of ultrasound treatment parameters and microbubbles dose will have been optimized.

## Figures and Tables

**Table 1:** Microbubble properties including composition, size distribution, and clinical dose. Data for mean diameters, concentration, gas volume and microbubble number were adapted from 32.

Type of Microbubble	Gas Core	Shell composition	Mean Diameter (Volume)	Mean Diameter (Number)	Initial concentration	Injection Gas Volume (typical injected dose)	Total Number Microbubble (typical injected dose)	Full Clinical Dose for Bolus Injection	Other info
Lumason	sulfur hexafluoride	phospholipid	8 µm	1.9 µm	3.4x10^8^ MB/mL	0.77 µg/kg	5.8x10^6^ MB/kg	34 µL/kg (2.4 mL/bolus for a 70-kg person	Marketed as SonoVue in Europe (Bracco Imaging SpA, Colleretto Giacosa, Italy)
Definity	perflutren (C_3_F_8_)	phospholipid	8 µm	1.2 µm	84x10^8^ MB/mL	1.65 µg/kg	12.6x10^6^ MB/kg	3 µL/kg (0.2 mL/bolus for a 70-kg person	Lantheus Medical Imaging, North Billerica, MA
Optison	perflutren (C_3_F_8_)	albumin	7 µm	3.1 µm	7.3x10^8^ MB/mL	1.65 µg/kg	5.5x10^6^ MB/kg	15 µL/kg, (1 mL/bolus for a 70-kg person	GE Healthcare AS, Oslo, Norway

**Table 2:** Summary of antivascular effect and other bioeffects

Effect	Mechanism
Platelet and thrombosis model	MB mechanically damages the endothelium exposing components of the basement membrane, which then causes platelet aggregation and subsequent vessel thrombosis.
Ceramide model (Radiosensitization)	MB-induced cavitation increases the amount of ASMase and ceramide production leading to endothelial cell death through perturbation of the cell membrane, and increased sensitivity to radiation.
Temperature model	Treatment with long pulse duty cycles results in local temperature elevation, arising from absorption of ultrasound energy by viscous damping of the oscillating MB.
Mechanical lesioning model	MB-induced cavitation leads to mechanically induced damage to the microvasculature and direct mechanical tissue lesioning, as well as indirect lesioning by ischemic necrosis.

**Table 3:** Antivascular tumors treatment

Ref.	Animal Model	Microbubbles (MB)	US Exposure Conditions	Biological Effects
				
108	Mice Murine melanoma (K1735) Subcutaneous	Definity, Bolus: 0.2mL Equivalent to 1.7 x10^9^ MB or 8.5 x10^10^ MB/Kg	Physiotherapeutic US machine (D150 Plus; Dynatronics Corp) 3 MHz Unfocused CW for 1 or 3 minutes SATA 2.3 W/cm^2^ Estimated pressure amplitude 0.22 MPa MI = 0.13	Significant decrease in the perfused area and in perfusion, treatment-time dependent. Mean decrease in perfusion area of 45% after 1mn of treatment, 67% after 3mn. Focal hemorrhage in area of decreased perfusion, dilated and thrombosed vessels. Presence of local inflammatory response, with increased infiltration of increasing HIF1A+ cells and CD45+CD3+ T cell infiltration in tumors. Tumor blood flow assessed with contrast-enhanced power Doppler (immediately before and after treatment) and Dynamic Contrast-Enhanced MRI (1d before, 5hr after treatment),
58	Mice Met-1 or NDL tumors Mammary fat pads	Lipid-shelled MB: targeted (integrin cyclic-RGD or LXY-3 peptide-conjugated) control non-targeted MB Size 2µm Bolus, 10^8^ MB Equivalent to 5.10^9^ MB/kg	Siemens Sequoia 15L8 transducer, 5 MHz color-Doppler pulses, 8.1 ms pulse repetition period, 6-cycle pulse length, 900 ms insonation 4 MPa or 2 MPa PNP MI = 1.8	Reduced regions of blood flow after destructive pulses applied to bound. No flow reduction with flowing MB. Decreasing pulse pressure (4 to 2 MPa) reduced the occurrence of regions of reduced blood flow. No histological changes in the tumor interstitium, no hemorrhage. Vasculature recovery within 30mn Tumor blood flow alteration assessed by CEUS CPS sequence, histology, platelets binding (50min after treatment)
62	Mice Murine colon adenocarcinoma (MC38) Subcutaneous	Lipid shell + perfluorobutane Diameter 2 µm Bolus: 2 injections separated by 10mn, 25 x10^8^ MB/Kg Equivalent to x100 Definity or x215 Sonovue clinical doses	Philips TIPS device F = 1.2 MHz 3 pulse trains of 10 pulses of 100,000, PRF = 1 Hz Pulse trains separated by a 20 second off period. PNP = 5MPa Effective treatment time = 2.5s (DC 3.5%)	Acute blood flow disruption nearly complete after a single treatment, blood flow returned after about 5-10 minute. Blood flow restoration makes unlikely that vascular endothelium lining were killed acutely, could be vasospasm. No temperature rise recorded during treatment Tumor growth almost completely stopped when daily treatment applied.
45	Mice Glioma C6 Subcutaneous	Lipid+ decafluorobutane Infusion, 10^8^ MB/kg in 0.3mL Equivalent to x1.5 Definity clinical dose (Infusion)	unfocused, 0.75-in-diameter transducer 1MHz 5 bursts of 5000 or 10000-cycles, separated by 50ms OFF Repeated every 5s For 60mn PNP = 1 or 1.2 MHz MI = 1 or 1.2 Effective sonication time = 18 or 36s (DC 0.5 or 1%)	Duty cycle-dependent tumor blood flow reduction immediately after treatment, with perfused area down to 4% (1% DC) of pretreatment flow. Duty cycle-dependent increase in intratumor temperature: +2.5°C for 0.5% DC, +5°C for 1% DC Tumor necrosis and apoptosis significantly increased post-treatment.
47	Mice nude Breast cancer (human, MDA-MB- Hind legs	Definity Bolus, repeated 3 times at 10min intervals 5x10^8^ MB/kg (60 µL/kg) Equivalent to x20 Definity clinical dose (single bolus)	focused transducer, 3.75 cm dimeter; 15 cm focal length 1MHz Bursts of 50 0.1 ms pulses, spaced 1 ms apart, repeated at 20s intervals For 3 min. PNP at focus 1.65 MPa MI = 1.65 Effective treatment time = 45ms	Acute reduction of perfusion, sustained 24h and 3days after the treatment. Significant growth inhibition following USMB treatment. Combination of antivascular USMB effects with an antiangiogenic therapy (Cyclophosphamide) showed significant growth inhibition and survival prolongation compared to USMB or drug alone. PCD recording shows that inertial cavitation is occurring during the treatment
48	Mice (nude) Prostate (Human, PC3) Subcutaneous	Experimental MB (Artenga, Ottawa, Canada) Octofluropropane in sorbitan monostearate (Span 60) and Tween 80 shell, diameter 2.13 µm Bolus, 2.1x10^5^ MB/g of mouse weight Equivalent to 2.1x10^8^ MB/kg	spherically focused transducer (diameter 3.75 cm; focal length 15 cm) 1 MHz 50 0.1 ms pulses, spaced 1 ms apart, repeated every 20s For 3 mn Repeated 3 times at 10mn intervals PNP 1.65 MPa	Significant 10-fold reduction of flow within the central regions of the tumors, not in the periphery Higher levels of necrosis and apoptosis at 24h Reduction in perfusion staining at 24h, more pronounced in central region of the tumor No improvement in tumor growth control when treatment with MB alone. Significant growth delay and improved survival when treatment with MB+docetaxel PCD recording (single element focused transducer, 750 kHz; focal length 7.5 cm, diameter 2.5 cm, confocally aligned with therapeutic transducer) show that during treatment, subharmonic, ultra-harmonic, and broadband noise are present, associated with MB stable oscillations and inertial cavitation.
110	Mice colon cell carcinoma (CT26.wt) Subcutaneous, hindlimb	Pegylated phospholipid shells + octafluoropropane (Artenga, Ottawa, ON, Canada) Mean diameter 1-3.7µm Bolus, 9.6x10^8^ MB/kg	focused transducer, 3.75 cm dimeter; 15 cm focal length 1MHz Bursts of 50 0.1 ms pulses, spaced 1 ms apart, repeated at 20s intervals For 2 min. Repeated once after 10min. PNP at focus 1.65 MPa MI = 1.65	Shutdown of blood flow and higher necrosis within the tumors after treatment with US+MB. Treatment with US+MB results in delayed tumor growth Treatment with US+MB and aPD1 results in smaller tumors, longer survival time. But absence of evidence by flow cytometry of a shift T-cell subpopulations to a more favorable anti-tumor state. PCD recording (focused 0.75- MHz transducer), show the presence of broadband emissions, a hallmark of inertial cavitation indicating the presence of violent microbubble oscillations during treatment
22	Rabbit Auricular vein	Optison Bolus, 0.5mL Equivalent to 7.3 x10^7^ MB/kg, or x7 Optison clinical dose (single bolus)	focused transducer, 34.9 mm diameter, 5cm focal 1.13 MHz Pulses of 500 cycles PRF 5 Hz (DC =0.22%) For 60s Effective treatment time 132 ms The pulsing sequence started immediately after IV injection of MB	Significant endothelial damage induced in vessels significantly larger (approximately 1 mm diameter) than capillaries Discernable damage (histology) confined to the luminal surface of the blood vessels. No sign of thermal damage to perivascular tissues.
44	Rabbit Auricular vein	Optison Bolus, 0.5mL Equivalent to 7.3 x10^7^ MB/kg, or x7 Optison clinical dose (single bolus)	focused transducer, 34.9 mm diameter, 5cm focal 1.13 MHz Pulses of 500 or 5000 cycles PRF 1HZ (DC 0.04% or 0.4%), For 1 to 120s The pulsing sequence started immediately after IV injection of MB	Significant endothelial damage, resulting in platelet adhesion to the endothelial surface and the formation of an intravascular fibrin thrombus, only in the presence of circulating UCA. Endothelial damage increased with increasing PNP. Higher IC doses correlated with greater endothelial damage TEM images consistent with a mechanical rather than a thermal mechanism of injury to the vascular wall 89
67	Mice Melanoma (K173522) Subcutaneous	Definity Bolus, 0.2mL / animal Equivalent to 8.5x10^10^ MB/Kg or x3400 Definity (single bolus)	Physiotherapy device (D150 Plus, Dynatronics Corp., Salt Lake City, UT, USA) 1 or 3 MHz CW For 3mn PNP =0.27 MPa	Overall reduction of tumor perfusion of 75% at 3MHz. Enhanced reduction of perfusion at 3MHz than 1 MHz. Predominant acute effects = dilation of the tumor capillaries and hemorrhage Temperature increase of 7.8 °C at 1 MHz and 15°C at 3 MHz Because MB were adminstered in bolus, and since the insonation time used in this study was 180s, it suggests that inertial cavitation may not have been a dominant factor in the observed antivascular effects. But rather relies on thermal effects
54	Mice Melanoma (K173522) Subcutaneous	Definity Bolus, 0.2mL / animal Equivalent to 8.5x10^10^ MB/Kg or x3400 Definity (single bolus)	Physiotherapy device (D150 Plus, Dynatronics Corp., Salt Lake City, UT, USA) 3 MHz CW Three 1-min treatments with a 1-min gap between each PNP =0.27 MPa Effective treatment time 3mn	Reduction in tumor growth rate and increased survival time. No monitoring of tumor perfusion changes
55	Mice Sarcoma (S-180) subcutaneous	Lipid-shelled perfluoropropane microbubbles Bolus, 1.5x10^8^ MB/animal Equivalent to 7.5x10^9^ MB/kg	Therapeutic device (KHT-017 transducer, DCT-700, Shenzhen Well.D Medical Electronic, Shenzhen, China). 0.94 MHz PRF 10Hz DC 0.19% 1 min (3s ON, 9s OFF) PNP 0.5, 1.5, 3.0, and 5.0 MPa	Significant decreased perfusion immediately after treatment, sustained at 24h. At 3 MPa, decrease by 84% of blood perfusion and microvessel density of the tumor at 24h. Promoted of tumor cell necrosis and apoptosis, delayed tumor growth, and increased survival rate of tumor-bearing mice
56	Mice (nude) Pancreactic (XPA-1-RFP) Subcutaneous	Targestar®-P (lipid encapsulated decafluorobutane) 1.9 or 2.9 µm diameter 1×10^8^ MB in 70 μl per mouse Equivalent to 5x10^9^ MB/kg	Sonicator, 1cm^2^ tip (Haiying Medical Electronic Instrument Company, Wuxi, China) 238kHz 10ms pulse length DC50% For 60s Repeated 3 successive days 0.5 MPa	Significant reduction in tumor growth compared to control, with both MB sizes. Treatment with 2.9µm MB resulted in more tumor cell necrosis and apoptosis, decreased expression of CD31 and micro-vessel density.
162	Mice Prostate (PC3) Subcutaneous	Perfluoropropane-albumin MB (Kangrui Pharmaceutical Co., Yueyang, Hunan, China) 3.4µm diameter Bolus Dose: 0.05, 0.10 or 0.20 ml at 6.5x108 MB/mL Equivalent to 1.6 to 6.5x10^9^ MB/Kg	3 low-frequency US systems (Shanghai Institute of Ultrasound in Medicine, China) 20, 80 and 500 kHz DC 20% (1s ON/4s OFF), 40 (2s ON/3s OFF), or 60% (3s ON/2s OFF) Intensity 0.50, 1.00 or 2.00 W/cm^2^ For 1, 3 or 5 min	All parameters tested, sound intensity, frequency, duty cycle, MB dose, treatment time influenced the decrease in perfusion, with optimal treatment parameters 1 W/cm^2^, 20 Hz, DC 40%, MB dose 0.20 ml, treatment time 3 min. Perfusion reduced immediately after treatment, with histology indicating disruption of vascular wall, mcrovessel dilation, edema in the vicini[58]f the ruptured vessels
109	Mice Colon carcinoma (CT-26) subcutaneous	Sonovue Bolus 0.1 mL/kg	Focused transducer (diam 64mm, focus 55mm) 0.5MHz burst length = 100 ms, PFR 1 Hz For 20s 9 to 12 sonications for coverage of the entire tumor PNP 0.6 or 1.4 MP (MI 0.84 or 2)	No apparent impact on perfusion, as quantified by CEUS after treatment. 2 hr MB treatment, significant increase in vessel permeability, enlarged vascular/cellular or extracellular spaces, with local erythrocyte extravasations, but no apparent increase in apoptotic cells. Significant decrease in tumor volume (18% at 0.6 MPa, and 34% at 1.4 MPa) compared to controls after 16d Local modulation of the immune environment: transient increase in infiltration of non-T regulatory (non-Treg) tumor infiltrating lymphocytes (TILs), continual infiltration of CD8+ cytotoxic T-lymphocytes (CTL), increased CD8+/Treg ratio. No temperature increase (measured in mimicking phantom using the same FUS exposure energy)
163	Rat Walker carcinoma (Walker 256) Subcutaneous	perfluoropropane encapsulated in lipid shell (DPPG, DSPE) mean diameter of 2 µm Bolus 0.04 mL at 9x10^10^/mL.	Focused transducer (25mm dimeter, focus 16cm) 831 KHz pulse length of 300cycles, PRF = 1Hz, 6s ON and 6s OFF (DC = 0.0019). Peak pressure: 2.6 MPa or 4.8 MPa	Blood flow circulation could be completely blocked off immediately after treatment, for 24 hours in tumors treated at 4.8 MPa, and with a slow recovery starting within 60mn at 2.6MPa Disruption of tumor microvasculature into diffuse hematomas accompanied by thrombosis, intercellular edema and multiple cysts formation. The 24 hours of tumor circulation blockage resulted in massive necrosis of the tumor.

The references included in the Table are representative of the different treatment schemes proposed to induce vascular flow disruption in tumors. When mentioned, equivalent dose in MB/kg were estimated assuming a 20g mouse. MI = Mechanical Index, PNP = Peak Negative Pressure, PRF = Pulse Repetition Frequency, DC = Duty Cycle, CW = Continuous Wave, SATA = Spatial Average Temporal Average

**Table 4:** Normal brain lesions

Ref.	Animal Model	Microbubbles (MB)	US Exposure Conditions	Biological Effects
				
49	Rhesus macaques Normal brains Targets near the skull base	Definity, Bolus: 20 µml/kg (equivalent to 2 times clinical imaging dose) Definity, Infusion: 0.1 ml/min for the first 10 seconds, 0.02 ml/min thereafter	ExAblate MRgFUS 220kHz (InSightec) 220KHz focused 10-msec bursts PRF 1Hz for 5 minutes. Pressure level typically 500kPa, either slightly above or below the cavitation threshold (as assessed by d broadband emissions)	When inertial cavitation present: localized ischemic necrosis, lesions formation with central region containing red blood cell extravasations surrounded by edema, presumably resulting from mechanically induced damage to the microvasculature BBB disruption in the lesions and prefocal area of the FUS system. With bolus injection, a strong inertial cavitation was observed at the start of sonication for about 10 seconds, and then low-level broadband activity With Infusion, a strong inertial cavitation observed was observed sporadically throughout the sonication, with strength of the low-level broadband signal increased over time
102	Rats Normal brain Trasncranial	Definity Bolus 10 or 20 μl/kg Equivalent to 0.85 or 1.7 x10^8^ MB or 3.5 or 7 x10^8^ MB/Kg	F = 525 kHz 10-msec bursts PRF 1 Hz for 5 minutes PNP estimated at 174 or 195 kPa MI = 0.24 or 0.27	Lesions produced via destruction of the vasculature and subsequent ischemia in downstream tissues. Damages limited to the endothelium. Ultrasound-induced damage appeared to preferentially affect gray matter structures, more vascularized. T1w imaging suggest that blood flow into the vessels was not stopped. Presumably, these lesions resulted from vascular damage and, in some cases, rupture produced by inertial cavitation, which led to reactive vasospasm, ischemia, and subsequent ischemic necrosis
98	Rabbit Brain Transcranial	Optison Bolus 0.05 mL/kg Equivalent to 3.65 x10^7^ MB/kg or x3.6 Optison clinical dose (single bolus)	Focused transducer, diameter, 10 cm; focal length 8 cm 1.5 MHz 500ms pulse length, PRF 1Hz, For 10 or 20s PNP 2-4MPa Effective treatment time 5 or 10s	Necrotic lesions, most likely result of cavitation-related damage, time-averaged power to induce lesions was less than one-tenth of what was needed to produce thermal lesions (without microbubbles) and peak temperature remained low. Changes in perfusion were not assessed.
101	Rat Brain Transcranial	Optison Bolus 100 µL/kg Equivalent to 7.3.10^7^ MB/Kg) or x6.6 Optison (single bolus)	FUS 1.1-MHz 10-msec bursts PRF 1 Hz For 300 seconds	Lesions evident immediately after each sonication, presence of microhemorrhages (T2*-weighted images). No delayed hemorrhages. Cystic lesions formed within 2 weeks after sonication stable over time. Results consistent with the timeline with tissue necrosis after ischemic stroke

The references included in the Table are representative of the different treatment schemes proposed to induce lesion in normal brain using destruction of microbubbles. When mentioned, equivalent dose in MB/kg were estimated assuming a 20g mouse. MI = Mechanical Index, PNP = Peak Negative Pressure, PRF = Pulse Repetition Frequency

**Table 5:** Radiosensitization: Antivascular effect combined with radiotherapy

Ref.	Animal Model	Microbubbles (MB)	US Exposure Conditions	Biological Effects
				
71	Rats (nude) Hepatocellular carcinoma Orthotopic	Optison Bolus 2.4 x10^8^ MB/Kg Equivalent to a x22 clinical dose of Optison (bolus)	Siemens S3000 scanner with 9L4 probe F= 4.2 MHz 1.6 ms pulses PRF 38 Hz for 4s. Repeated 4 times per tumor plane, for a total treatment time of 2-3mn PNP = 2.5 MPa MI = 1.35 Effective treatment time = 0.92s (DC 3%)	Linear decrease of tumor vascularity when the number of destructive sequences increased from 0 to 3. 67% decrease in tumor vascularity after 3 destructive pulses No increase in tumor hypoxia 3 h post treatment (photo-ascoutic assessment). When combined with radiotherapy (5Gy single dose), 3hrs after MB treatment, significant improvements in survival time in animals compared to single modality treatment (US of RT), demonstrating a sensitization of tumors to RT.
79	Mice Fibrosarcoma (MCA-129) Hind leg	Definity Injected during treatment (5mn) 25 µL or 75µL	500kHz 16-cycle tone burst PRF 3 kHz 5 min PNP 500kPa (MI 0.8)	With MB treatment alone, no significant effect on tumor perfusion, microvascular density, ISEL and ceramide expression at 3, 24, 72 hrs post MB treatment. After radiation, single dose 2 or 8Gy, significant acute reduction in blood flow at 8Gy 3hr after treatment Combined treatment MB destruction and RT (immediately after MB treatment) at 8Gy results in almost 50% decrease in tumor perfusion, peaking at 24h and persisting for up to 72hrs, accompanied by extensive tumor cell death. Use of genetic and chemical approaches demonstrates the role ASMase-ceramide pathway in mechanotransductive vascular targeting using USMB, driving an[56]anced radiation response.
70	Mice (SCID) Prostate (human PC3) Subcutaneous	Definity 3.6x10^8^ or 1.08x10^9^ MB (100- and 300-fold diagnostic dose). VEGFR2 targeted MB	Focused single element 500kHz 10% duty cycle within a 50-ms window every 2 s total active insonification time of 750 ms over 5 min for an overall duty cycle of 0.25%. 570kPa (MI 0.76)	Endothelial cell apoptosis induced by MB treatment, and enhanced when combined with RT, leading to a reduction in blood flow and the induction of tumor cell death MB treatment alone maximal effect on blood flow and cell death after 6 h. Greater effect when using VEGFR2-targeted MB MB effects diminished by protection of the vasculature with bFGF Significant reduction of blood flow to tumor treated with the MB destruction and radiotherapy in combination Radiation therapy combined with MB treatment (high concentration) showed effective tumor growth delay at 20d (8Gy single treatment, or 2Gy multiple fraction treatment). Greater survival when MB treatment combined with RT at multiple fraction, with significant effects at non-curative RT dose Involvement of ceramide cell death pathway.
75	Mice, wild-type or asmase knockout (ko) Fibrosarcoma MCA-129 Hind leg	Definity Infusion 25 µL or 77 µL / mice Equivalent to 10^10^ MB/Kg or 3x10^10^ MB/Kg x71 Definity (Infusion) X205 Definity (Infusion) Clinical doses	F= 500 kHz 16-cycle tone burst, PFR 3 kHz (DC 1%) For 5 min PNP = 500 kPa MI = 0.71 Equivalent effective treatment time = 3s	No statistical effect on tumor perfusion as quantified with 3D power Doppler, with even a trend although non-significant of increased perfusion at 24h with the highest MB concentration used. But decrease in tumor perfusion when US MB destruction combined with radiotherapy treatment. Proposed mechanisms : mechanotransductive vascular targeting of ASMase-ceramide pathway, causing EC death when combined with radiation.

The references included in the Table are representative of the different treatment schemes proposed to enhance response to radiotherapy via blood flow disruption following microbubbles destruction. When mentioned, equivalent dose in MB/kg were estimated assuming a 20g mouse. MI = Mechanical Index, PNP = Peak Negative Pressure, PRF = Pulse Repetition Frequency, DC = Duty Cycle
